# Improving glaucoma staging in clinical practice by combining the ICD-10 glaucoma severity classification system and optical coherence tomography

**DOI:** 10.1038/s41433-023-02650-5

**Published:** 2023-06-30

**Authors:** Ari Leshno, Emmanouil Tsamis, Noga Harizman, Carlos Gustavo De Moraes, Sol La Bruna, Anvit Rai, Aakriti Garg-Shukla, George A. Cioffi, Qing Wang, Jeffrey M. Liebmann, Donald C. Hood

**Affiliations:** 1 Bernard and Shirlee Brown Glaucoma Research Laboratory, Edward S. Harkness Eye Institute, Department of Ophthalmology, Columbia University Irving Medical Center, 635 W 165th St, New York, NY 10032 USA; 2 Sackler Faculty of Medicine, Tel Aviv University, Tel Aviv, Israel; 3 Department of Psychology, Columbia University Schermerhorn Hall, 1190 Amsterdam Ave #406, New York, NY 10027 USA; 4 Albert Einstein College of Medicine, New York, NY 10461 USA

**Keywords:** Glaucoma, Optic nerve diseases

## Abstract

**Objective:**

The International Classification of Disease, 10th revision (ICD-10) codes used for glaucoma severity classification are based on the 24-2 visual-field (VF) test. This study aim was to assess the added value of providing clinicians with optical coherence tomography (OCT) data, in addition to functional data, for glaucoma staging in clinical practice.

**Exposure:**

Disease classification was determined for 54 glaucoma eyes, according to the principles of the ICD-10 guidelines. Eyes were independently graded in a masked fashion using the 24-2 VF test and 10-2 VF test, with and without OCT information. The reference standard (RS) for severity was determined using a previously published automated structure-function topographic agreement for glaucomatous damage using all available information.

**Results:**

The RS classified eyes as mild, moderate and advanced in 3, 16 and 35 cases, respectively. Individual and combined 24-2 and 10-2 based gradings were significantly different from the RS (all *P* < 0.005), with Kappa agreements of 0.26, 0.45 and 0.42 respectively (*P* < 0.001). Classifications using OCT combined with either of the VF were not-significantly different from the RS (*P* > 0.3) with Kappa agreements of 0.56 and 0.57 respectively (*P* < 0.001). Combining 24-2 with OCT had less severity overestimations while 10-2 with OCT had fewer underestimations.

**Conclusion:**

Combining OCT and VF data provides better staging of glaucoma severity than VF data alone. The 24-2 and OCT combination seems most appropriate given the high concordance with the RS and less overestimation of severity. Incorporating structural information into disease stages allows clinicians to set more appropriate severity-based treatment targets for individual patients.

## Introduction

**Fig. 1:**
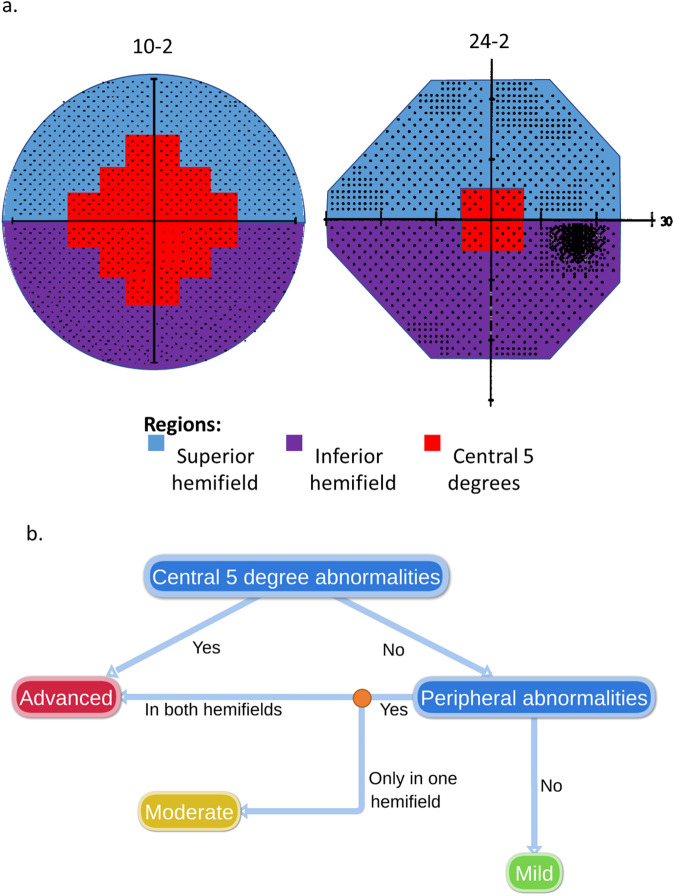
The rules of the International Classification of Diseases, 10th revision (ICD-10) glaucoma severity reference guide. **a** Based on the ICD-10 glaucoma severity reference guide, in order to define severity, the entire visual field (VF) is divided into three regions: superior hemifield (blue), inferior hemifield (purple), and central 5 degrees of fixation (red). **b** Based on which regions are involved, the guide dictates the severity: abnormalities within the central region classify an eye as Advanced regardless of the number of hemifields involved. If the center is not involved then severity is defined based on the number of hemifields with abnormalities: both hemifields = Advanced; one hemifield = Moderate; none = Mild.

The International Classification of Diseases (ICD) is widely accepted as a classification system for clinical, research and health management purposes for medical conditions, including glaucoma. In addition to specifying glaucoma diagnosis (e.g, open-angle vs angle-closure glaucoma, primary vs. secondary mechanisms, etc.), the current 10th revision (ICD-10) also includes classification of disease severity. The classification requires the presence of “optic nerve abnormalities consistent with glaucoma,” as a necessary condition for the diagnosis, however severity is determined based solely on abnormalities found within three regions on the visual field (VF) test: superior hemifield, inferior hemifield and central 5 degrees (Fig. 1a) [1].

There are several important advantages to the regional classification method of the ICD-10. First, compared to other VF-based classification methods (e.g., the Hodapp-Anderson-Parrish criteria and Brusini Enhanced Glaucoma Staging System), the rules are fairly simple to understand and relatively easy to apply. In the ICD-10 system, eyes with normal visual function are recorded as mild, involvement of both hemifields or loss within the central 5 degrees are classified as advanced, and the remaining eyes with field loss are classified as moderate. It is worth noting that the classification gives special consideration to the central 5 degrees due to the importance of central vision for daily activity and quality of life [2–4]. The same is true for other VF-based glaucoma classification systems, (e.g. HPA, Glaucoma Visual Field Staging System [GVFSS]). However, this region is covered by only 4 points in the 24-2 and 30-2 perimetry tests, which limits the ability of these tests to detect abnormalities in this region.

In a previous study [5], we applied the same set of rules as the ICD-10 to structural information obtained by optical coherence tomography (OCT) and found a wide range of structural damage within each ICD-10 VF-based severity group. There was also a significant overlap in the extent of structural damage across the VF-based severity groups. Furthermore, in over 40% of the eyes, the OCT detected central damage missed by the 24-2. These findings coincide with previous studies showing that classification systems that relies exclusively on the 24-2 or 30-2 VF are likely to underestimate severity by missing central damage [6–8]. In that study we hypothesized that by adding OCT information to the VF, this grading system would result in a more accurate reflection of the extent of glaucomatous damage.

The aim of the current study is to assess whether combining OCT and VF data provides better staging of glaucoma severity than VF data alone and to determine which functional test (24-2 or 10-2) is best for this purpose.

## Methods

### Subjects

This retrospective cross-sectional study was approved by the institutional review board for human research of Columbia University Irving Medical Center and followed the tenets of the Declaration of Helsinki and the Health Insurance Portability and Accountability Act. Written informed consent was obtained from all eligible participants.

Data collected from patients who participated in a previous clinical study (the Macular Damage in Early Glaucoma and Progression Study; PI: C Gustavo De Moraes; ClinicalTrials.gov Identifier: NCT02547740) were used. All eyes had refractive error between –6 and +6 diopters. Fifty-four eyes for which a wide-field swept-source OCT scan (12 x 9 mm, Topcon, Inc., Paramus, NJ, USA), involving both the macula and the optic disc, color disc photo, 24-2 visual field (VF) and 10-2 VF (Swedish Interactive Testing Algorithm–Standard, Humphrey Field Analyzer; Carl Zeiss, Meditec, Inc., Dublin, CA, USA) were available within a 6-month time frame were included. The presence of GON in all cases was confirmed by two independent glaucoma experts (CGD, QW) based on all available data. If more than one VF was available, the first reliable test (false positive rate ≤15% and fixation losses ≤ 33%) was used. The OCT was performed within 6 months of the 24-2 VF (mean 29.9, median [IQR] 0 [0-3.5] days).

### ICD-10 severity classifications

The ICD-10 glaucoma severity reference guide [1] defines severity based on the extent of regional involvement on the VF. In particular, the entire VF is divided into three regions: superior hemifield, inferior hemifield, and central 5 degrees (Fig. 1a). Severity is defined according to the presence of abnormalities within these three regions with: “Mild” indicating that none of the regions are involved; “Moderate”, only one of the hemifields is involved without the central region; and “Advanced”, either both hemifields are involved and/or the central region is involved.

The ICD-10 glaucoma severity definition (mild, moderate, advanced) was determined for each eye by a separate group of two independent graders (NH, JML). The graders had five rounds of severity classification. In the first two rounds, they were presented with either a 10-2 or a 24-2 VF test. For the remaining three rounds, the following combinations of tests were presented: 24-2 VF with OCT, 10-2 VF with OCT, and 24-2 VF with 10-2 VF. In each round, the graders were asked to define regional involvement (superior/inferior hemifield and central 5 degrees) based on the presented information, and to determine severity according to the rules of the ICD-10 glaucoma reference guide for each eye.

Disagreements were resolved after a discussion between the two graders. In each round, the graders were masked to all other data, including the original classification in the medical chart or any gradings from previous rounds.

### Objective automated structure-function reference standard (RS)

**Fig. 2:**
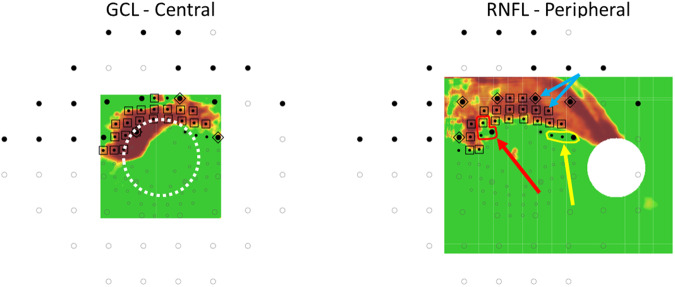
The combined structure-function probability map. Data from the pattern deviation maps of both 10-2 and 24-2 VF as well as the RNFL and GCL probability maps (presented in field view) were combined to generate an objective measure for regional involvement that served as the reference standard. The 10-2 (small dots) and 24-2 (large dots) VF points are superimposed over the corresponding topographic locations on the RNFL and GCL probability maps. The black-filled points indicate that the pattern deviation was below the 5% threshold. Structure-function (S-F) agreements are indicated by the black outline surrounding the points (square for the 10-2 points and diamond for the 24-2 points, blue arrows), using a 10% threshold for the RNFL and GCL thickness. The rules used to determine involvement for each of the regions, based on this combined S-F probability map, are described in detail in the methods section.

Because the ICD-10 does not provide specific details as to what should be considered an abnormality within each region, the classification, regardless of the test it is based upon, is subjective and open to interpretation. Therefore, we modified a previously described method for automated detection of topographic agreement to create an objective measure for regional involvement [9]. We used that modified approach as the reference standard (RS) for the extent of the disease. In particular, an automated structure-function report was generated using combined data obtained from the pattern deviation maps of both the 10-2 and 24-2 VF, and the RNFL and GCL probability maps (see Fig. 2). Abnormal locations for the 24-2 and 10-2 VF (threshold probability <5%) are shown as large and small black-filled circles respectively, and the abnormal RNFL and GCL regions are color-coded yellow-red (threshold probability <10%). Based on our previous work [9], a location was considered abnormal if it was confirmed by at least two of the 3 tests (i.e., OCT, 24-2 VF and 10-2 VF). We defined three types of abnormal locations. First, when both the OCT region and the superimposed VF location were abnormal, it was called “abnormal structure—abnormal function (aS-aF).” An aS-aF location based on a 24-2 and 10-2 VF point is demarcated with a diamond and square respectively in Figure B. Second, a cluster of abnormal VF points, located within one of the ICD-10 regions (i.e., superior hemifield, inferior hemifield, or central 5 degrees), and comprised of at least 3 adjacent points taken from both the 10-2 and 24-2 VF (within the same region) was called an “abnormal function—abnormal function (aF-aF)” region. Third, if at least one of the abnormal VF points within an aF-aF location was marked as an aS-aF location, it was considered an “abnormal structural component (aF-aF-aS) location.”

For each eye, regional involvement (i.e., superior hemifield, inferior hemifield, central) was automatically classified based on a set of predetermined rules. In particular, a region was defined as “involved” if it satisfied at least one of the following criteria:

1. Two aS-aF locations within the same region (Fig. 2. blue arrows)
2. Two aF-aF locations within the same region (Fig. 2. yellow arrow and outline)
3. A combination of one aS-aF location and one aF-aF location within the same region.
4. One point of aF-aF-aS location (Fig. 2. red arrow and outline)
5. Peripheral structural defects were based only on the RNFL probability map
6. Central structural defects were defined based only on the GCL probability map. In addition, central structural damage was only considered to be abnormal if accompanied by a corresponding RNFL arcuate defect.

A summary of the mean number of abnormal points for each of the regions involved according to the RS is presented in eTable 1.

The regional decisions were translated to an ICD-10 equivalent severity score based on the flow-chart in Fig. 1b. Of the 54 eyes, 3 were mild, 16 moderate, and 35 advanced.

### Statistics

The statistical software SPSS version 25.0 (SPSS,Inc., Chicago, IL, USA). A p-value of <0.05 was considered significant. Accuracy rates and Cohens Kappa were calculated to estimate the agreement of each of the proposed grading methods to the RS. Marginal homogeneity test was also applied to determine if the gradings differed significantly from the RS.

## Results

Table 1 summarizes the glaucoma experts’ agreement with the RS gradings when the experts used either a single VF test, a combination of both VFs, or a combination of one type of VF with the OCT. A detailed comparison between each of the grading methods to the RS is available in the supplementary material (eTable 2).

**Table 1:** Agreement of gradings methods with RS.

Method	Kappa	Accuracy	Under-estimation	Over-estimation	*P* value*
24-2 only	0.258 ± 0.097	30 (55.6%)	19 (35.2%)	5 (9.3%)	0.002
10-2 only	0.452 ± 0.097	37 (68.5%)	15 (27.8%)	2 (3.7%)	0.002
24-2 with 10-2	0.416 ± 0.101	36 (66.7%)	15 (27.8%)	3 (5.5%)	0.004
**24-2 with OCT**	**0.562** **±** **0.101**	**42 (77.8%)**	**7 (13.0%)**	**5 (9.3%)**	**0.346**
**10-2 with OCT**	**0.574** **±** **0.107**	**43 (79.6%)**	**4 (7.4%)**	**7 (13.0%)**	**0.808**

^*^ Marginal Homogeneity test comparing the severity decisions by each grading method to the RS.

The gradings based on a combination of either the 24-2 or the 10-2 with the OCT had the best overall performance (bold in Table 1). Close to 80% of the eyes were graded with the same severity as the RS (red in eTable 2), and based on the marginal homogeneity test, both were not significantly different from the RS (*P* value > 0.34). These two methods differed mainly in the direction of misclassification: gradings based on the 24-2 & OCT combination tended to have more cases of underestimation of severity, whereas the gradings based on the 10-2 & OCT combination tended to have more cases of over-estimation of severity (Table 1). An example for each is available online (eFig. 1).

**Fig. 3:**
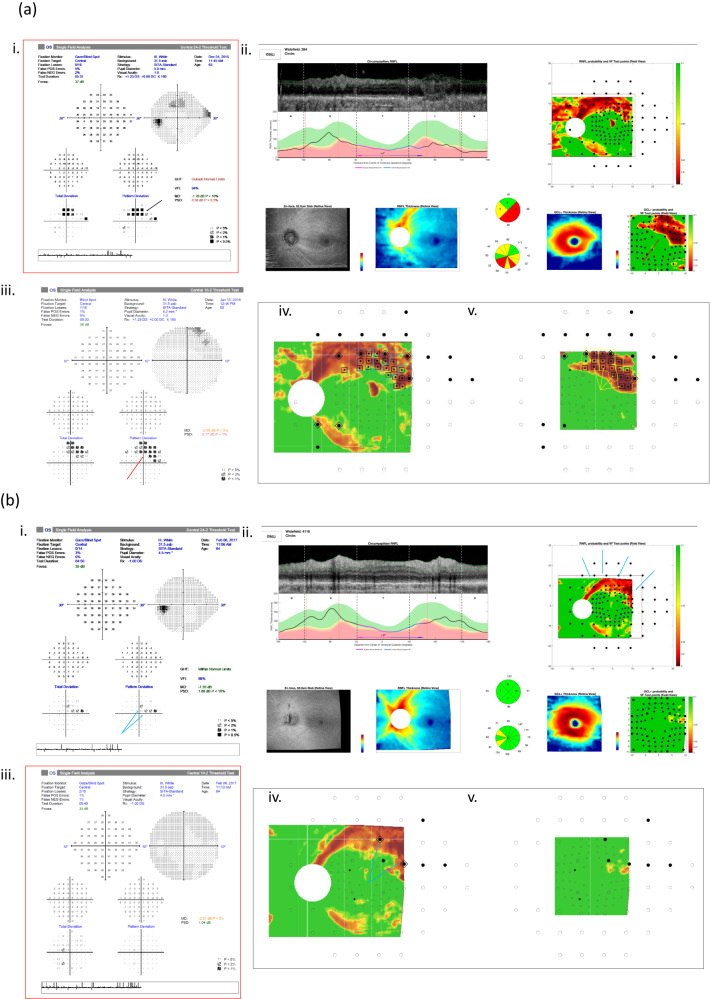
Under-estimation of severity by a single visual field test due to missed central (a) or peripheral (b) involvement. **a** An example of the gradings based on the 24-2 under-estimating severity due to missed central involvement. Based only on the 24-2 (i. red rectangle) the graders classified severity as moderate due to superior hemifield involvement (black arrow). However, according to the RS (iv and v—black rectangle), this eye had superior hemifield involvement on the RNFL probability map (iv) based on 24 aS-aF locations and 4 aS-aF-aF clusters (blue outline), and central involvement on the GCL probability map (v) based on 4 aS-aF locations (yellow arrows) and 3 aS-aF-aF clusters (yellow outline) within the central 5 degrees. Therefore, the RS severity was classified as advanced. Note that when the graders evaluated the severity for this eye based on the 10-2 (iii) alone as well as based on the combination of OCT (ii) with either 24-2 or 10-2, they agreed with the RS and classified the severity as advanced due to central involvement, in agreement with the damage on the GCL probability map (red arrows). **b** An example of the gradings based on the 10-2 under-estimating severity due to missed hemifield involvement. Based only on the 10-2 (iii, red rectangle) the graders classified severity as mild. However, according to the RS, this eye had superior hemifield involvement on the RNFL probability map (iv) based on 2 aS-aF locations from the 24-2 pattern deviation map, in agreement with the superior arcuate defect on the RNFL probability map (blue arrows). Therefore, the RS severity was classified as moderate. Note that when the graders evaluated the severity for this eye based on the 24-2 (i) alone as well as based on the combination of OCT (ii) with either 24-2 or 10-2, they agreed with the RS and classified the severity as moderate due to superior hemifield involvement.

The gradings based on the 24-2 and 10-2 VF tests, whether alone or in combination were significantly different from the RS (marginal homogeneity test *P* value < 0.004). This was largely due to underestimation of the severity, which occurred in approximately a third of the cases. Missed central involvement was the most common cause for underestimation of severity with gradings based only on the 24-2 (13 out of the 19 cases of underestimation) as shown in the example in Fig. 3a. On the other hand, over half (8 out of 15) of the underestimations of the 10-2 based gradings were related to missed hemifield involvement, as shown in the example in Fig. 3b.

The combination of 24-2 and 10-2 provided poorer agreement with the RS than the 10-2 alone, although the agreement was better than the gradings based only on the 24-2. Again, most of the disagreements with the RS (15 out of 18 cases) were due to underestimation of severity. This was due to missed central involvement in 8 cases and missed involvement of at least one hemifield involvement in 7 cases. An example for each type of underestimation is available online (eFig. 2).

**Fig. 4:**
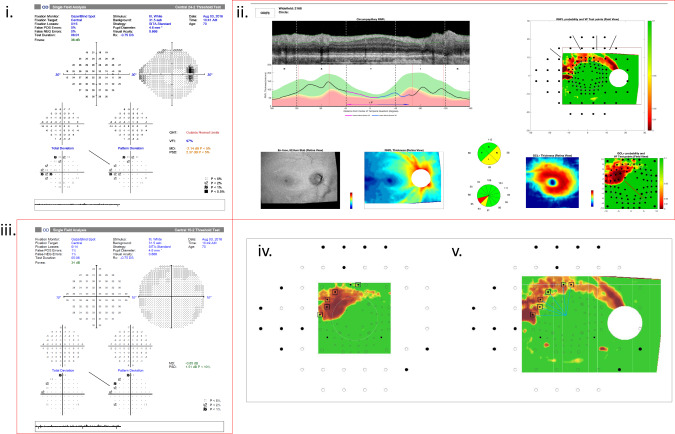
Accurate estimation of severity by the 10-2 alone but over-estimation when combined with OCT. An example case of when the gradings based on the 10-2 accurately estimated severity, however the combination of 10-2 with OCT over-estimated the severity. In this eye, according to the RS (black rectangle) there was superior hemifield involvement on the RNFL probability map (v) based on 6 aS-aF locations (blue arrows) and no locations that met the threshold criteria for central 5-degree involvement (white circle) on the GCL probability map (iv). Therefore, the RS severity was classified as moderate. Based on the combination of the 10-2 with the OCT (iii and ii respectively, red rectangles) the graders classified severity as advanced, due to the superior RNFL arcuate (black arrows) extending to the central 5-degree as seen on the GCL probability map (red arrows). However, based only on the 10-2 visual field the graders classified the severity of this eye as moderate due to superior hemifield involvement (black arrow), in agreement with the RNFL defect and the RS decision.

When the gradings were based on the 24-2 alone, severity was overestimated, compared to the RS, in 5/54 (9.3%) cases. All of these were due to hemifield involvement that was considered significant by the graders based on the 24-2 but did not meet the RS threshold. With the addition of the OCT, the graders agreed with the RS regarding the hemifield involvement in 3 out of the five cases. However, the 10% overestimation rate remained unchanged when the gradings were based on the combination of 24-2 with OCT, due to a greater number of cases that were considered to have central involvement. The lowest rate of overestimation was for the gradings based only on the 10-2 (2/54, 3.7%). However, the overestimation rate became much higher with the addition of OCT to the 10-2 (7/54, 13%). An example of such a case of overestimation is shown in Fig. 4.

## Discussion

The purpose of this study was to assess the added value of providing clinicians with structural information (i.e., OCT), in addition to functional information (i.e., 10-2 and 24-2 VF) to better stage glaucoma severity based on the rules of the ICD-10. We found that when using only functional data, the graders tended to underestimate severity as compared to an objective assessment of severity (i.e., the RS), even when both the 10-2 and 24-2 were provided for the grading. As we hypothesized, a better agreement with the RS was achieved when the graders’ severity decisions were based on combined OCT and VF information.

### The limitation of using only functional information

Although the importance of VF testing in glaucoma and its substantial association with measures of vision-related quality of life has been described extensively [2–4, 10–14], VF tests have several limitations and each VF pattern has its own drawbacks. The 24-2 test pattern covers most of the functional visual field, but has low central spatial resolution [6, 7, 15–21]. Consequently, grading severity based only on the 24-2 will underestimate severity due to missed central damage as evident in our results. An example of such a case is provided in Fig. 3a. This an important limitation to the ICD-10 classification, which typically uses the 24-2 test, and thus often underestimate the presence and extent of macular damage, the region that is crucial for performance of day-to-day activities. Various studies have shown that both structural and functional glaucomatous macular damage correlate with poor contrast sensitivity, facial recognition and overall diminished vision related quality of life [4, 14, 22–25]. Given the low sensitivity of the 24-2 to central damage, significant damage that impacts quality of life can be easily missed.

Conversely, the 10-2 pattern has relatively high central spatial resolution, but covers a limited area of the visual field (less than a quarter of the area of the 24-2) and will miss more peripheral hemifield involvement. In addition, the limited field makes it harder to identify the arcuate patterns of visual field defects that start outside the central 10°. An example of this underestimation of severity due to missed peripheral damage, is shown in Fig. 3b.

The joint use of the two types of VF patterns did not provide a better outcome than using the 10-2 alone, and improved the accuracy of the gradings using the 24-2 by only 6 cases. In other words, the advantages of having more peripheral or central VF information, did not significantly aid in grading severity when compared to one type of VF test alone. This is likely due to the fact that both tests are based on the same principles and share similar sources for inherent variability. In addition, the relatively small spatial overlap between the two tests, especially in the central 5 degrees, limits the ability to of each of the tests to confirm the other. In any case, in the clinical setting it is usually impractical to obtain both a 10-2 and a 24-2 on the same visit due to time constraints and testing fatigue.

### The power of combining structure and functional information

The best performance involved a combination of either the 10-2 or the 24-2 with the OCT. The advantage of these combinations is probably due to the VF and OCT tests having unrelated sources of variability, making artifacts more easily distinguished from real damage. An example is available online (Fig. S-1a).

Adding the OCT to the disease severity classification system compensates for the limitations embedded in each of the VF patterns. The OCT and specifically the GCL maps offer high spatial resolution for the central region, which helped the graders to reduce the rate of missed central involvement (as determined by the RS) when using only the 24-2. The added value of the OCT is also supported by previous studies that found a correlation between structural macular damage to functional vision and vision related quality of life [4]. In addition, the wide field RNFL scan covers a large enough area that compensates for the relatively narrow area of the 10-2 and helps identify the arcuate pattern. In addition, using information from OCT is also likely to improve the accuracy of severity classification in patients with visual field tests with low-reliability.

In any case, both the 10-2 and 24-2 are roughly equivalent as functional tests for glaucoma severity grading when combined with the OCT. Both combinations provided a similar degree of accuracy (Kappa value close to 0.6) and statistically were not different than the RS. While the 10-2 is useful in clinical management, it is not required for staging when the OCT is incorporated into the classification alongside the 24-2. Assuming that a classification system must be highly specific, and avoid overestimation of severity in order to be effective, the combination of the OCT with the 24-2 has the advantage in having lower rates of overestimation than the OCT combined with the 10-2. The trade-off is that some cases will have a severity classification that underrepresents the extent of the glaucomatous disease. From a clinical perspective, misclassifying a patient as being worse than they actually are might lead to an unnecessarily aggressive or higher-risk intervention. Conversely, while inadvertently staging a patient into a lower-risk category may result in undertreatment, subsequent testing will likely provide additional staging and progression information that will alter the periodicity of surveillance or treatment intensity. This is especially true for identifying central involvement due to the significant impact on quality of life.

### Limitations

Even the best combination of OCT and VF tests agreed with our RS in only 80% of the eyes. It is important to note that the RS used herein was not intended as a gold standard for determining glaucoma severity, but rather to provide an objective and standardized measure to test the ICD-10 rules. The empirical set of rules of determining regional involvement for the RS was derived from previously published data [9], and included information from all three tests in a way that treated them as equally as possible. Modifying our RS rules or thresholds might affect the decisions on regional involvement and consequently the agreement of the various grading methods with the RS on severity. However, it should be noted that in most cases the number of abnormal points far exceeded the threshold for determining regional involvement (Table S1). It might also be argued that the 24-2 is under-represented by our RS, as most of the field points have no spatial agreement with either the 10-2 or the OCT image because of the scan field size. However, the fact that the 24-2 based grading had very few cases of over-classification indicates that this was not the main cause for disagreements with the RS.

While our data confirm the limitations of the current ICD-10 system and suggest that structural data needs to be integrated to enhance the disease classification and staging rubric, additional research is needed to help develop more precise guidance for the clinician to maximize diagnostic accuracy. An ideal classification method should be as objective as possible, preferably automated, to ensure standardization and repeatability. While the automated S-F agreement used here as a reference standard could be an adequate first step in this direction, it has some limitations. Using the algorithm requires both 24-2 and 10-2 visual field tests, in addition to an OCT scan. While the combination of 24-2 and 10-2 VF provides a more comprehensive evaluation of functional damage [8], it is not always practical to perform both tests on the same visit. In addition, although the use of OCT in glaucoma management is growing, it might not be as readily available as perimetry, which may limit the applicability of our approach in certain regions. The RS might also be less useful among individuals that are not well represented by the OCT normative database (e.g., high myopes). In such cases the presence of artifacts in the probability (aka deviation map) of the OCT, due to mere anatomic variability, can increase the rates of false-positive or false-negative results for regional involvement. However, the spatial agreement between the OCT and VF required by the RS minimizes that risk. Given that the variance of each test is independent and related to their unique normative database, the probability of yielding a false positive or false negative result in the same location on both tests is highly unlikely (due to the Multiplication Rule of Probability for independent events). Regardless, we encourage clinicians and researchers to always carefully review the complete report to exclude any artifacts that may affect the grading. Notwithstanding these shortcomings, more accurate staging of disease severity will aid physician decision-making and the development of health care policy to more clearly focus on those individuals at greatest risk for a decrease in vision-related quality of life or blindness. Further validation is needed to determine the relationship between quality-of-life measures and combining OCT and VF to determine regional damage. Future considerations could include objective and, preferably automated, alternatives to the ICD-10 that maintain its intuitive basis and generalizability across clinical practices. The ICD classification should be revisited when OCT becomes more widely available worldwide.

## Summary

Combining OCT and VF data provides better staging of glaucoma severity than VF data alone. The 24-2 and OCT combination seems most appropriate given the high concordance with the S-F RS and lowest rate of overestimation of severity. Incorporating structural information into disease stages allows clinicians to set more appropriate severity-based treatment targets for individual patients.

## Summary

### What was known before

- The ICD-10 glaucoma severity classification system is usually based on the 24-2 visual fields which have low sensitivity to central damage and therefore can result in underestimation of disease severity

### What this study adds

- This cross-sectional study of 54 eyes demonstrated that combining optical coherence tomography and visual field data improves the accuracy of disease severity estimation and staging compared to the use of functional testing alone, as defined by the ICD-10 classification system.

### Supplementary information

Supplemtal material

## Data Availability

Data is available and would be provided upon reasonable request.
