# Supplementary material for: Improving glaucoma staging in clinical practice by combining the ICD-10 glaucoma severity classification system and optical coherence tomography
Source: Eye (Lond). 2023 Jun 30;38(1):153–60. doi: 10.1038/s41433-023-02650-5 (PMC10764715; doi:10.1038/s41433-023-02650-5)
Supplement: Supplementary file 1 — Supplemtal material [file 41433_2023_2650_MOESM1_ESM.docx]

| **eTable 1. Summary of mean±SD number of abnormal points for defining a region as involved based on the RS** | | | |
| --- | --- | --- | --- |
|  | **aS-aF points** | **aF-aF points** | **aS-a-F-aF points** |
| **Superior hemifield** | 11.8±10.1 | 2.8±2.7 | 2.8±2.7 |
| **Inferior hemifield** | 9.3±10.7 | 2.4±2.2 | 2.1±2.3 |
| **Central 5 degrees** | 7.1±5.3 | 1.8±1.2 | 1.8±1.2 |

**eTable 2. Comparison between gradings methods and the RS.**

|  | **24-2** | | | **10-2** | | |  |
| --- | --- | --- | --- | --- | --- | --- | --- |
| **RS** | **Mild** | **Moderate** | **Advanced** | **Mild** | **Moderate** | **Advanced** | **Grand Total** |
| **Mild** | 2 | 1 | 0 | 3 | 0 | 0 | 3 |
| **Moderate** | 5 | 7 | 4 | 6 | 8 | 2 | 16 |
| **Advanced** | 6 | 8 | 21 | 3 | 6 | 26 | 35 |
| **Grand Total** | 13 | 16 | 25 | 12 | 14 | 28 | 54 |

|  | **Combined 10-2 & 24-2** | | |  | |
| --- | --- | --- | --- | --- | --- |
| **RS** | **Mild** | **Moderate** | **Advanced** | **Grand Total** |  |
| **Mild** | 3 | 0 | 0 | 3 |  |
| **Moderate** | 5 | 8 | 3 | 16 |  |
| **Advanced** | 3 | 7 | 25 | 35 |  |
| **Grand Total** | 11 | 15 | 28 | 54 |  |

|  | **24-2 & OCT** | | | **10-2 & OCT** | | |  |
| --- | --- | --- | --- | --- | --- | --- | --- |
| **RS** | **Mild** | **Moderate** | **Advanced** | **Mild** | **Moderate** | **Advanced** | **Grand Total** |
| **Mild** | 2 | 1 | 0 | 2 | 1 | 0 | 3 |
| **Moderate** | 3 | 9 | 4 | 1 | 9 | 6 | 16 |
| **Advanced** | 2 | 2 | 31 | 2 | 1 | 32 | 35 |
| **Grand Total** | 7 | 12 | 35 | 5 | 11 | 38 | 54 |

| eFig. 1. Examples of misclassification of severity when based on the combination of the OCT with (a) 24-2 or (b) the 10-2 visual fields. |
| --- |
| (a)  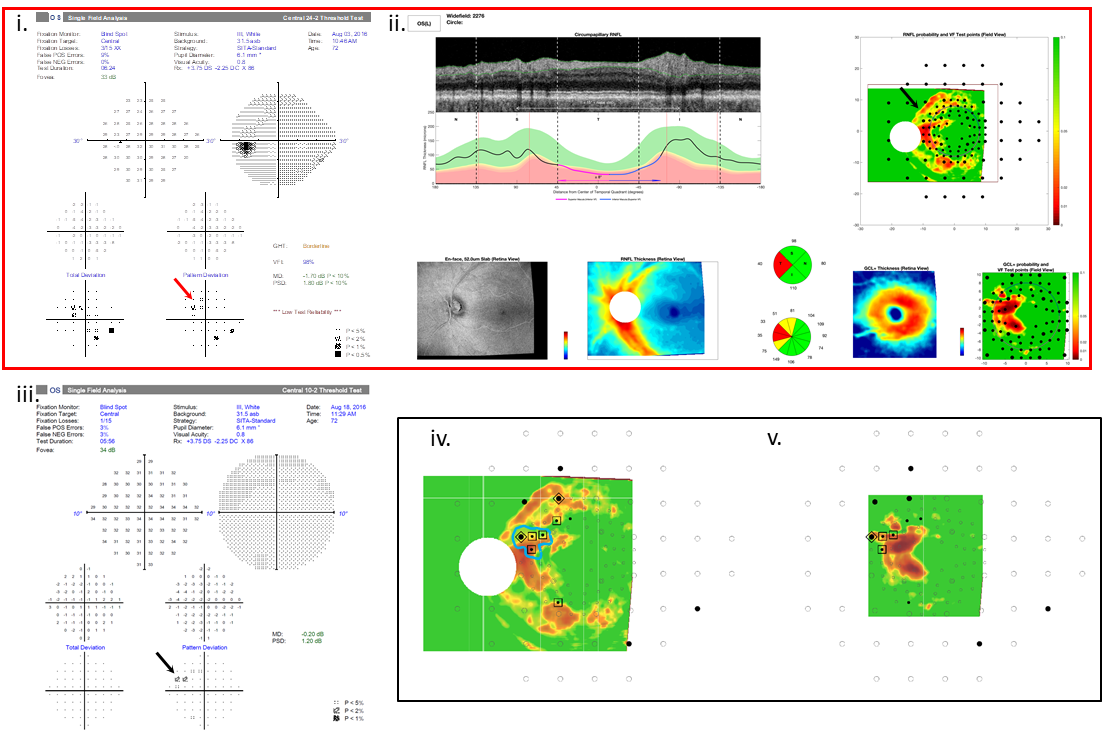 |
| (b)  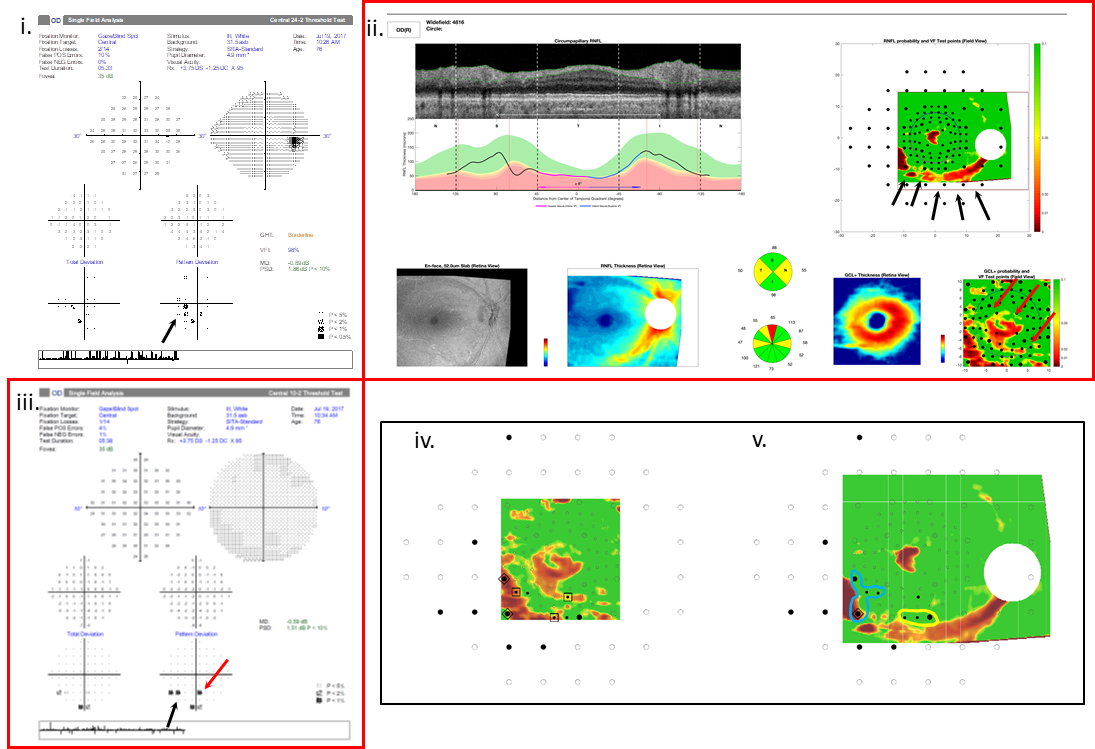 |
| (a) An example of under-estimation of severity with gradings based on the combination of the 24-2 visual fields (i) with the OCT report (ii). Based on this combination (within red rectangle), the graders classified the glaucoma severity of the eye as mild (i.e., no regional involvement). However, according to the RS (iv and v, black rectangle), there was superior hemifield involvement based on 6 aS-aF locations (black diamond and square demarcations) and one aS-aF-aF cluster (blue outline) on the RNFL probability map (iv). Note that when the graders evaluated the severity for this eye based on the combination of the 10-2 (iii) with the OCT, it was classified as moderate due to superior hemifield involvement (black arrow), in agreement with the RS. A closer look at the 24-2 pattern deviation map shows spatial agreement with the 10-2 and OCT (red arrow).  This is also a good example of the advantage of combining VF and OCT information. Note that there is a small cluster of abnormal points on the superior-temporal quadrant of the 10-2 (iii) that does not seem significant in itself – likely why the grades classified the eye as mild based on the 10-2. The RNFL probability map looks abnormal, however it does not have the expected pattern of glaucomatous damage. However, combining the information derived from both tests (iv) shows spatial agreement for damage in the superior hemifield, explaining why the graders decided to classify this eye as moderate based on the 10-2 and OCT combination. Note that although a similar spatial agreement exists between the 10-2 and the 24-2 (blue demarcation on v and black arrows on i and iii) this was not considered significant by the graders, who classified the eye as mild based on the combination of these two functional tests. This might be in part because of the assumption that the functional testing variability might account for the same artifacts on both the 24-2 and the 10-2 test results.  (b) An example of over-estimation of severity with gradings based on the combination of the 10-2 visual fields (iii) with the OCT report (ii). Based on this combination (red rectangles), the graders classified severity of the eye as advanced due to inferior hemifield involvement (black arrows) that also includes the central 5 degrees (red arrows). However, according to the RS (black rectangle), this eye had only inferior hemifield involvement based on 1 aF-aF cluster (yellow outline) and 1 aS-aF-aF clusters (blue outline) on the RNFL probability map (v), but it did not meet the threshold for central involvement (iv). Therefore, the RS severity was classified as moderate. Note that when the graders evaluated the severity for this eye based on the 10-2 alone (iii), the 24-2 alone, or on the combination of OCT with the 24-2 VF (i), they agreed with the RS and classified the severity as moderate due to inferior hemifield involvement. |

| eFig. 2. under-estimation of severity by the 24-2 & 10-2 combination due to missed (a) hemifield (a) and central (b) involvement |
| --- |
| (a)  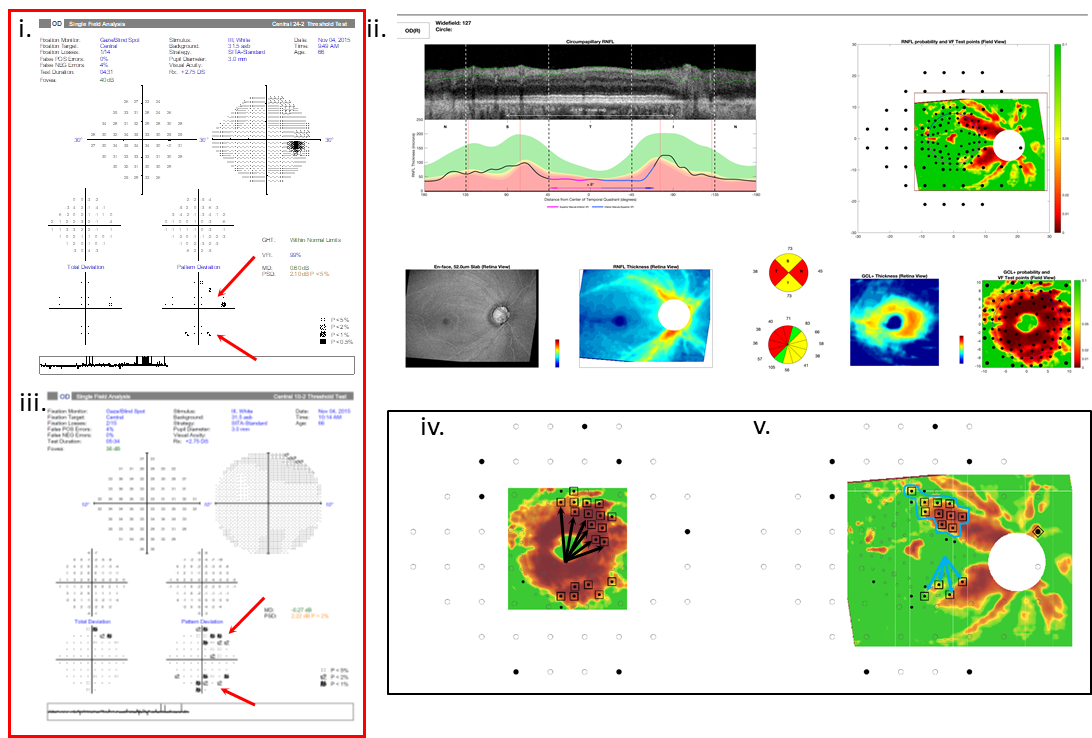 |
| (b)  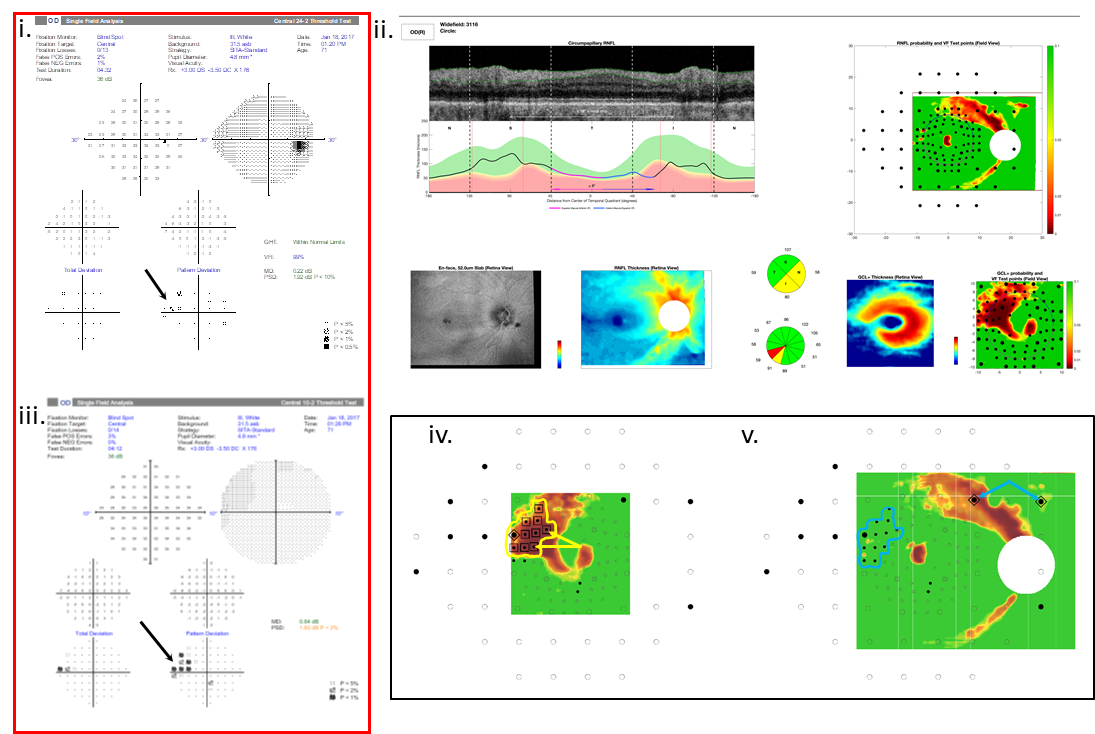 |
| Two example cases of when the gradings based on the 24-2 & 10-2 combination under-estimated severity. (a) According to the RS (iv and v - black square), this eye had involvement of both hemifields on the RNFL probability map (v) based on 10 aS-aF locations in the superior hemifield (blue outline) and 4 aS-aF locations in the inferior hemifield (blue arrows). In addition, it had central involvement on the GCL probability map (iv) based on 6 aS-aF locations within the central 5 degrees (black arrows). Therefore, severity was classified as advanced. Conversely, when the graders evaluated the severity for this eye based on the combination of the 10-2 (iii) and 24-2 (i) (red square) they defined the severity as mild, presumably considering the abnormalities on the VF (red arrows) as artifacts.  (b) according to the RS (iv and v – black square) the superior hemifield was considered involved on the RNFL probability map (v) based on two aS-aF locations (blue arrows) and a aF-aF cluster (blue outline). Central 5 degrees involvement on the GCL probability map (iv) was based on two aS-aF locations (yellow arrows) and a aS-aF-aF cluster (yellow outline). Conversely, based on the combination of the 10-2 (iii) and 24-2 (i) (red square) the graders defined the severity as moderate, based on involvement of the superior hemifield (black arrows) without involving the central 5 degrees. Note that in both (a) and (b), when the graders evaluated the severity for this eye based on the 10-2 alone as well as based on the combination of OCT (ii) with either 24-2 or 10-2, they agreed with the RS and classified the severity as advanced. |
